# Association of first trimester maternal vitamin D, ferritin and hemoglobin level with third trimester fetal biometry: result from cohort study on vitamin D status and its impact during pregnancy and childhood in Indonesia

**DOI:** 10.1186/s12884-019-2263-1

**Published:** 2019-04-02

**Authors:** Raden Tina Dewi Judistiani, Tita Husnitawati Madjid, Setyorini Irianti, Yessika Adelwin Natalia, Agnes Rengga Indrati, Mohammad Ghozali, Yunia Sribudiani, Tetty Yuniati, Rizky Abdulah, Budi Setiabudiawan

**Affiliations:** 1 Public Health Department- Centre of Immunology Studies, Faculty of Medicine Universitas Padjadjaran, Jl. Raya Bandung Sumedang KM.21, Kecamatan Jatinangor, Kabupaten Sumedang, Jawa Barat 45363 Indonesia; 2 Obstetrics and Gynecology Department, Faculty of Medicine Universitas Padjadjaran, Sumedang, Indonesia; 3 dr Hasan Sadikin Hospital, Bandung, Indonesia; 4 Clinical Pathology Department, Faculty of Medicine Universitas Padjadjaran, Sumedang, Indonesia; 5 Department of Basic Medical Sciences, Faculty of Medicine Universitas Padjadjaran, Sumedang, Indonesia; 6 Department of Child Health, Faculty of Medicine Universitas Padjadjaran, Sumedang, Indonesia; 7 Department of Clinical Pharmacology, Faculty of Pharmacy Universitas Padjadjaran, Sumedang, Indonesia

**Keywords:** Fetal growth, Ferritin, Haemoglobin, Maternal vitamin D, Pregnancy

## Abstract

**Introduction:**

The role of vitamin D in placental functions and fetal growth had been addressed in many reports with conflicting results. However, such report is limited for Indonesian population. The aim of this study was to explore the association between maternal vitamin D level in the first trimester and fetal biometry in the later stage of pregnancy with adjusted OR for other determinants like hemoglobin and ferritin level.

**Methods:**

From July 2016 a prospective cohort study of pregnant women had begun in four cities in West Java, Indonesia. Data on maternal vitamin D, ferritin, hemoglobin level, maternal demography and fetal biometry were analyzed with linear regression.

**Results:**

Among 203 recruited women, 195 (96.06%) had hypovitaminosis D. One hundred fifty two (75%) were in deficient state and 43 women (21%) were in insufficient state. Women with insufficient vitamin D had the highest proportion of anemia, while women with normal vitamin D level had the highest proportion of low ferritin level. Maternal serum vitamin D showed significant associations with biparietal diameter (β = 0.141, *p* = 0.042) and abdominal circumference (β = 0.819, *p* = 0.001) after adjustment with maternal age, pre-pregnancy body mass index, parity, serum ferritin level, and hemoglobin level.

**Conclusion:**

Our study suggested that sufficient maternal vitamin D level was an important factor to improve fetal growth and development.

## Background

Maternal hypovitaminosis D was increasingly associated with a higher incidence of fetal miscarriage, preeclampsia, gestational diabetes, bacterial vaginosis, and impaired fetal and childhood growth and development [1]. Fetal growth and development is highly influenced by maternal nutrition state before and during pregnancy. In general, the practice of healthy and balanced diet should have started a long time prior to conception and be maintained all through the journey of any pregnancy.

One of many important micronutrients for fetal growth is vitamin D, a fat soluble vitamin produced by the skin of human body with the energy received from ultraviolet B exposure [2]. Dietary intake may not be enough, although vitamin D can also be obtained from certain foods such as fatty fish, mushrooms, egg yolks, liver and dairy products [3, 4].

A study has shown that vitamin D’s main role is in muscle, bone and mineral homeostasis especially related to calcium regulation [5]. Several in vitro studies demonstrated genomic effects of vitamin D receptors in muscle differentiation, bone mineralization and muscle fiber size [6, 7]. Other study confirmed that vitamin D was an important regulator for osteoblast activity in various skeletal sites [8].

Within the last 27 years attention toward the role of vitamin D in human life increased for its wider role in health maintenance and disease prevention; recommendations for different vitamin D levels have been proposed for different health problems, diseases, across age and sex. Vitamin D supplementation in combination with calcium, had been found beneficial in the prevention of bone fracture among the elderly [9], moreover vitamin D fortified cheese consumption proved to prevent bone resorption in elderly [10].

Low intake of vitamin D during pregnancy was associated with asthma in children and supplementation during pregnancy reduced the risk of asthma or recurrent wheeze during the first three years of life [11]. The Cochrane review on vitamin D supplemetation for women during pregnancy found some indication that vitamin D supplementation could reduce the risk of preeclampsia, increase length and head circumference at birth, but it also suggested further rigorous randomized trials were required to confirm these effects [12]. Supplementation of vitamin D among women with normal vitamin D level in early pregnancy was also found beneficial to prevent recurrent preeclampsia among women with history of preeclampsia (absolute risk reduction in the intervention arm 14.9%, p 0.036) [13]. Unfortunately neonatal outcomes were not assessed in this latest study. Today, it is widely known that pregnant women are in high risk to develop vitamin D deficiency or insufficiency. It has become a significant public health problem around the world, especially in South Asia and Middle East region [14]. Several factors influence vitamin D status in pregnant women such as skin pigmentation, adiposity status, geographical residence, dietary intake, ethnicity, and use of vitamin supplements [15–18].

Some studies showed that a higher proportion of women than men developed hypovitaminosis D in United States, Canada and some European countries [18–21]. Vitamin D deficiencies were prevalent among South Asian population in the United Kingdom, the figures reached 92%, the median of vitamin D level was as low as 9 ng/mL in summer and dropped to 5.8 ng/mL in winter [18].

Holick proposed to classify vitamin D status based on cut-off value, i.e. deficiency if 25-hydroxyvitamin D level was less than 20 ng/ml, the level of 21 to 29 ng/ml indicated a relative insufficiency and the level of 30 ng/ml or greater considered as sufficient [22].

Data on vitamin D status in Indonesia are scarce. The first report of vitamin D status among pregnant women was from a study in West Java which found that only 3.5% of pregnant women in their first trimester had normal cholecalciferol level (> 30 ng/mL), 21% had insufficient vitamin D (20–29 ng/mL) and the remaining 75.5% were deficient (< 20 ng/mL) [23]. Women with vitamin D deficiency in the first trimester were more likely to develop anemia in the third trimester [23].

Ferritin level was correlated with the presence of anemia [23] which can be explained by its function as the indicator for total iron reserve for hematopoiesis [24]. In addition, ferritin is also known as an important angiogenic factor to enhance tissue growth including the bone [24].

The effect of vitamin D during pregnancy is very important, but its effect on fetal anthropometric measurements remains unclear. Several studies reported inconsistent associations between maternal vitamin D levesl in pregnancy and risk of adverse pregnancy outcomes in different population, such as fetal growth pattern, preterm birth and small for gestational age [25–28].

Given the high prevalence of vitamin D deficiency during pregnancy and how it influenced fetal growth pattern, the identification of a link between maternal vitamin D level and intrauterine fetal growth measurements became important. One study in multiethnic population of Singapore did not find any associations between maternal low vitamin D level (below 30 nmol/L, equal to 50 ng/mL) and any of the birth outcomes which were being measured [29]. The lower cut-off point for vitamin D deficiencies below 20 ng/mL had been set earlier by Hollick to measure its impact on health problem [30]. While being very similar to Singapore in terms of geographical area, the knowledge on maternal vitamin D levels and its association with intrauterine growth remains limited in Indonesia. Therefore, this study aimed to explore the association between maternal vitamin D level in the first trimester and intrauterine fetal biometry in the later stage of pregnancy. Other determinants such as maternal characteristics, ferritin, and hemoglobin level were also calculated for adjustment.

## Methods

Data were taken from prospective cohort of pregnant women in four cities of Bandung, Cimahi, Waled, and Sukabumi, West Java, Indonesia. Potential participants were approached by community midwives and referred to study site hospitals, i.e. Dr. Hasan Sadikin Hospital and Kota Bandung Hospital (Bandung), Cibabat Hospital (Cimahi), Samsudin SH Hospital (Sukabumi) and Waled Hospital (Cirebon). Complete information regarding procedure of this study was given second time by a team of midwives, who had been recruited as our research assistants. Ultrasonography examinations were performed by obstetricians for fetal biometry and well-being at each referral hospital. Recruitment aimed at consecutively gathering 70–75 participants from each study site.

Pregnant women were eligible if they were (1) resident of the city, (2) willing to comply to the whole study procedure, (3) in between 10 and 14 weeks of pregnancy as confirmed by ultrasonography at recruitment, and (4) having a singleton, alive, normal fetus. Fetuses with congenital abnormalities were excluded from the study. After giving written consent, each participant was interviewed for demographic data, obstetric history and knowledge on nutrition support in pregnancy by research assistants. The participants were asked to fill a three-day food record within the following week and a diary on life style related to activity under sun exposure. Follow up examinations on fetal growth by ultrasound were carried out between 20 and 24 and 30–34 weeks of gestations. All records were kept as individual case report file in data storage for later entry.

In each visit, blood was drawn from median cubiti veins and examined for complete blood count and serum preparation. The complete blood count analysis was performed using automated hematology analyzer with impedance method measurement (Sysmex XP-100, Japan). The separated serum was stored at − 20 degree Celsius, before being transported in cool box to Dr. Hasan Sadikin Hospital for vitamin D and ferritin analysis. Serum vitamin D and ferritin analysis was performed by ELISA. The minimum measurable level of vitamin D in serum was 8.1 ng/mL; any level below that was reported as 8 ng/mL. Using Hollick’s parameter on vitamin D, subjects were classified into three groups (1) deficient (< 20 ng/mL); (2) insufficient (20–29.99 ng/mL); and (3) normal ≥30 ng/mL). Based on ferritin level parameter, the subjects were classified into normal (> = 30 ng/mL) or deficient (< 30 ng/mL).

Fetal ultrasonography was carried out once in each trimester (week 10–14, 20–24 and 30–34) to obtain data on fetal biparietal diameter, head circumference, abdominal circumference, and femur length. The assessments were performed by attending obstetricians who had already followed standardized ultrasonography training prior to the study. All measurements were compared to the Hadlock’s standard chart for intra uterine growth at the corresponding gestational age, to identify intra uterine growth restrictions,

The main analysis was performed using R 3.3.1. statistical software. Descriptive statistics were presented as frequencies and proportions for categorical variables, mean and standard deviation for continuous variables. Linear regression model was used to assess the association of maternal vitamin D level and fetal ultrasonography measurement in the third trimester. We included ferritin level, hemoglobin level, maternal age, parity, and pre-pregnancy body mass index as these variables might be associated with maternal vitamin D level [31–33]. Interactions between vitamin D and ferritin level, between vitamin D and hemoglobin level, and between ferritin and hemoglobin level were examined by adding interaction terms into the models. Results are presented as linear regression coefficients (β) and OR and 95% CI. *P*-value below 0.05 was considered statistically significant.

Ethics approval was given by Health Research Ethical Committee of Faculty of Medicine, Universitas Padjadjaran. Participation was based on informed written consent.

## Results

From July 2016 until March 2018, a total of 294 pregnant women had been recruited. Since the parent cohort study is still ongoing, only 203 participants were followed up until they delivered and thus included for this report. The characteristics of our study participants were shown in Table 1.

**Table 1:** Maternal and fetal characteristics of the study participants

Maternal characteristics	n (%)	Mean (SD)
Age (years)		28.78 (5.82)
Pre-pregnancy body mass index (kg/m^2^)		23.07 (5.16)
Parity		
0	69 (34)	
1	78 (38.4)	
≥ 2	56 (27.6)	
Serum vitamin D in the first trimester^a^		15.65 (7.06)
Deficient (< 20 ng/mL)	152 (0.75)	
Insufficient (20–29.99 ng/mL)	43 (0.21)	
Normal (≥ 30 ng/mL)	8 (0.04)	
Serum ferritin in the first trimester^b^		66.95 (53.48)
Hypoferritinemia (< 30 ng/mL)	68 (33.5)	
Normoferritinemia (≥30 ng/mL)	135 (66.5)	
Hemoglobin in the first trimester^c^		12.8 (1.98)
Anemia (< 11 g/dL)	15 (7.4)	
Normal (≥11 g/dL)	188 (92.6)	
Gestational age at the third trimester ultrasonography (week)		31.67 (1.56)
Biparietal diameter (mm)		79.82 (7.44)
Head circumference (mm)		282.3 (32.84)
Abdominal circumference (mm)		268.6 (23.32)
Femur length (mm)		63.56 39.47)

^a^Holicks’s parameter on vitamin D status [22]

^b^WHO parameter on iron status in population [54]

^c^WHO parameter for anemia in pregnancy [55]

In general, pregnant women in this study were in healthy reproductive age period, although 43 women (21.18%) were in high risk age group (< 20 years old or > 35 years old). Most nutritional status as defined by pre-pregnancy body mass index was within normal value. However, 32 women (15.76%) were in underweight group and 21 women (10.34%) were in obese group based on WHO classification [34]. A larger proportion of pregnant women who were underweight were at higher risk of anemia as a result of chronic undernourishment than those who were obese.

**Fig. 1:**
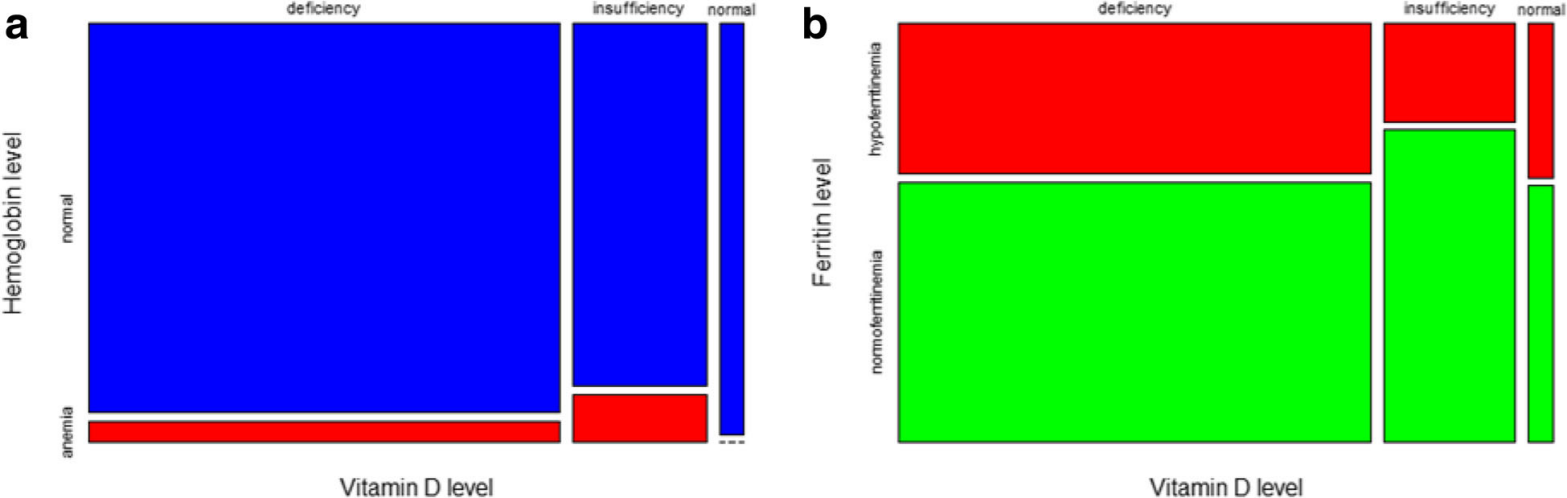
Distribution of vitamin D status according to hemoglobin level (**a**) and ferritin level (**b**) in the first trimester

At inclusion in the first trimester, maternal serum vitamin D values ranged from 8 ng/mL to 43.6 ng/mL. Around 195 women (96.06%) had hypovitaminosis D (< 30 ng/mL), in which 152 (75%) were deficient and 43 (21%) were insufficient. Women with insufficient vitamin D had the highest proportion of anemia, which may indicate that the presence of anemia was related to more factors other than vitamin D level. Women with normal vitamin D level had the highest proportion of low ferritin level (< 30 ng/mL) as shown in Fig. 1. It may be due to the small number of the subjects who had normal vitamin D (8 person) as compared to other groups, that another (case control) study may be needed to find their associations.

Fetal anthropometric data were analyzed from 176 subjects in the third trimester, but seven participants failed to return for follow up visit in the second trimester, thus leaving 169 data available for analysis. Results from linear regression were shown in Table 2. In univariable analysis, maternal serum vitamin D was significantly associated with biparietal diameter (β = 0.172, *p* = 0.028) and abdominal circumference (β = 0.819, *p* = 0.001). Maternal serum vitamin D still showed significant association with biparietal diameter (β = 0.157, *p* = 0.045 and β = 0.141, *p* = 0.042) and abdominal circumference (β = 0.828, p = 0.001 and β = 0.819, p = 0.001) after adjustment with maternal age, pre-pregnancy body mass index, parity, serum ferritin level, and hemoglobin level. However, after testing for interaction, only biparietal diameter was significantly associated with maternal vitamin D level (β = 1.255, *p* = 0.024), while serum ferritin and hemoglobin values demonstrated a significant interaction (β = − 0.015, *p* = 0.013). The prevalence of hypovitaminosis D in pregnant women was very high and around 75% of them were in deficient state of vitamin D (below 20 ng/mL). The result of this study could also reflect the real situation in the whole population, as it could be attributable to low intake of vitamin D-rich food and most likely change of life style with less outdoor activity. Some studies also reported that low intake of vitamin D and having no supplement or fortified food were reported in India, Saudi Arabia, and Japan [35–37].

**Table 2:** Associations between maternal serum vitamin D (treated as a continuous variable) in the first trimester with fetal biometry

Fetal characteristics	n	Univariable			Model 1^a^			Model 2^b^			Model 3^c^		
		β	95% CI	*p*-value	β	95% CI	*p*-value	β	95% CI	*p*-value	β	95% CI	*p*-value
Biparietal diameter													
Maternal serum vitamin D	176	0.172	0.019,0.325	0.028*	0.157	0.003,0.310	0.045*	0.141	0.005,0.278	0.042*	1.255	0.166,2.344	0.024*
Head circumference													
Maternal serum vitamin D	169	0.519	−0.171,1.208	0.140	0.457	−0.231,1.145	0.192	0.447	−0.336,1.230	0.261	1.219	−5.251,7.689	0.710
Abdominal circumference													
Maternal serum vitamin D	176	0.819	0.349,1.288	0.001**	0.828	0.360,1.297	0.001**	0.819	0.326,1.312	0.001**	− 0.449	−4.512,3.613	0.827
Femur length													
Maternal serum vitamin D	176	0.255	−0.566,1.076	0.541	0.255	−0.575,1.085	0.545	0.197	−0.782,1.175	0.692	1.678	−6.480,9.837	0.685

*p < 0.05

**p < 0.001

^a^Adjusted for maternal age, pre-pregnancy body mass index, and parity (treated as a continuous variable)

^b^ As model 1 with additional adjustment for serum ferritin and hemoglobin level

^c^As model 2 with interaction terms

For countries located at tropical region, the whole year availability of sunshine was considered a benefit. Ultraviolet B radiation is the primary source of energy to convert cholesterol into pre-vitamin D in the skin. The skin should have a direct contact to ultraviolet B, so it would highly be dependent on the daily outdoor activities, the skin color and life style of the people themselves, ranging from clothing style up to the use of sun protection cream. Brown to darker skin had a lower rate of pre-vitamin D synthesis even with the same duration of UVB radiation exposure as to those with lighter skin. The lower rate of pre vitamin D production came from the protective effects of melanin against UVB radiation [15, 18, 38, 39]. Interestingly, many women in Indonesia prefer to have brighter-fair skin and they purposefully wear sun protection cream to counter the ultraviolet B effect, even after knowing the beneficial effect of it. During the prime time to have the highest benefit from UV B radiation, from 10 am to 1 pm, most of the study subjects spent only 10 to 30 min per day in average.

The use of vitamin D supplementations or food fortification was recommended in food such as milk, butter and cereals, but the comsumption rate of such fortified food was low. The current practice of supplement dispensing during prenatal care in Indonesia were varied, but the basics were on iron, folic acid and calcium. However, it was also reported that concurrent use of vitamin D and calcium supplementation increased the risk of preterm birth [12].

Strong associations were found between maternal serum vitamin D level with some fetal anthropometric measurements, i.e. biparietal diameter and abdominal circumference, separately and after adjustment. Only a few studies investigated the association between maternal serum vitamin D and intrauterine fetal growth such as in Korea and Spain, in which no associations were found [40, 41]. Other studies were more focused on the pregnancy outcome and neonatal anthropometric measurements such as birth weight, birth length, head circumference, ponderal index, and mid-upper arm circumference with similar negative results [28, 29, 42]. Several systematic reviews found a positive association between maternal serum vitamin D and neonatal anthropometric measurements although a recent Cochrane review suggested inconclusive results [43–45].

In this study no associations was found between maternal vitamin D with fetal head circumference and femur length. As calcium and vitamin D were two important aspects of growth, the fetus depends solely on the supply through placenta, i.e. actively transporting calcium, magnesium, and some part of vitamin D from the maternal circulation through putative calcium-sensing receptors and other pathways, to set the fetal or placental transfer rate of nutrition at its high target value [46, 47]. In relation to vitamin D transfer, placental vitamin D receptor (VDR) expression in human placenta has been reported to be associated with neonatal 1,25(OH) vitamin D and 25(OH) vitamin D level. This indicated that placental VDR expression may be also involved in placental calcium transfer and thus fetal femur length and other parameters [48, 49]. The negative results were already observed in other studies [40, 41], another possible explanation was that it could also be attributable to the participation rate, availability of laboratory data on the third trimester at the time of data collection.

The association between maternal vitamin D and fetal biometry changed after interaction terms were included. Model 3 in Table 2 showed that only biparietal diameter showed significant association, which means that for any increase of vitamin D would also increase biparietal diameter. On the other hand, abdominal circumference was no longer associated to maternal vitamin D. Another interesting finding was that among the three interaction terms, only serum ferritin and hemoglobin level showed statistically significant result in the second model. Maternal anemia, as indicated by low hemoglobin concentration during pregnancy increased the risk of fetal adverse outcome like intra uterine growth retardation [50]. Aside from hemoglobin level, serum ferritin level could also be used as an indicator to evaluate maternal anemia status [51]. It may reflect maternal capability to increase erythropoiesis, together with the presence of vitamin D may also influence iron metabolism and erythropoiesis [52, 53]. Iron is also essential for vitamin D synthesis [52, 53]. Changes in the linear regression coefficient after addition of serum ferritin, hemoglobin level, and interaction between those two factors suggested similar effect. Further studies should be conducted to assess which of these indicators exerts a stronger influence over the other.

The strength of the present study lies on its population-based design and multiple locations that represented several geographical areas of West Java. Limited funding hindered this study to recruit more participants and postponement of investigation and analysis from blood samples taken in the second and third timester, as it may explain the correlation between vitamin D and ferritin changes in every trimester toward bone growth.

Negative findings on head circumference and femur length can be partially explained by postponement of investigation and analysis from blood samples taken in the second and third timesters as well as the placenta.

## Conclusion

This study showed that maternal vitamin D level was associated with fetal biparietal diameter and abdominal circumference at the third trimester; however, we found limited evidence for the association of maternal vitamin D level with head circumference and femur length. This suggested that sufficient maternal vitamin D level was an important factor to improve fetal growth and development. The possibility of interaction among vitamin D, ferritin, and hemoglobin level during pregnancy warrants further studies.
